# GABA Immunoreactivity and Pharmacological Effects vary Among Stylet-Bearing Nematodes

**DOI:** 10.2478/jofnem-2023-0049

**Published:** 2023-11-18

**Authors:** Hannah M. Reed, Ziduan Han, Nathan E. Schroeder

**Affiliations:** Department of Crop Sciences, University of Illinois at Urbana-Champaign, IL; College of Plant Protection, Northwest A&F University, Yangling, China; Neuroscience Program, University of Illinois at Urbana-Champaign, IL

**Keywords:** antibody staining, behavior, nematode control, neural anatomy, plant-parasitic nematode

## Abstract

Plant-parasitic nematodes conduct a series of sophisticated behaviors to complete their life cycles. Among these, locomotion behaviors, including finding the host and migrating to the feeding site, directly affect the success of parasitism. Thus, disrupting locomotion behaviors has the potential to control these parasites. γ-Aminobutyric acid (GABA) is the prominent inhibitory neurotransmitter in nematodes. GABA-immunoreactive neurons are mostly found in motor neurons, where they regulate behaviors in the model nematode *C. elegans*. However, the GABA system in most stylet-bearing nematodes has received little attention. Using immunohistochemistry, we found variation in the pattern of GABA-immunoreactivity among two major plant-parasites and a fungal feeder. Some of these GABA-immunoreactive neurons lack clear homologs to *C. elegans*. Pharmaceutical assays showed that applying GABA, its agonist, and its antagonist, can disrupt the locomotion behaviors of these nematodes, although sensitivity to a given compound varied between species. Our data suggest that the GABA system is a potential target for the control of plant-parasitic nematodes.

The conventional control of plant-parasitic nematodes relies heavily upon nematicides. Those chemicals often have a broad effect on animals, causing safety issues with humans and livestock and raising environmental concerns (Chitwood, 2003). Disrupting the regulation of behaviors of plant-parasitic nematodes through neuromuscular circuits has shown potential as a novel control strategy. Most parasitic nematodes are soil-borne and conduct a series of tasks to complete their life cycles. They live in the soil and migrate to a specific location in the roots of the host plant to feed and reproduce.

Many plant-parasitic nematodes display diverse behaviors, but effective locomotion behaviors are essential for their survival. For example, the migratory endoparasitic root-lesion nematodes (*Pratylenchus* spp.) live in the root tissues and do not have a fixed feeding site, and they can migrate into a new host plant during their life cycle (Duncan and Moens, 2013); both cyst and root-knot nematodes become immobile in the host once they begin feeding. In addition, during infection, root-knot nematodes migrate between cells to find their eventual feeding site, while cyst nematodes move intracellularly within the root tissue (Turner and Subbotin, 2013; Karssen et al., 2013).

In recent years, there has been a growing interest in developing nematicides with neuronal targets that disrupt nematode behaviors. In contrast to conventional nematicides, including organophosphates and carbamates, which act as acetylcholinesterase inhibitors (Chen et al., 2020), these chemicals may attack targets that are more nematode-specific. For instance, the ivermectin-family nematicide abamectin (Avicta), acting through the glutamate-gated chloride channels (Laing et al., 2017), was developed as a seed treatment for the control of plant-parasitic nematodes (Jensen et al., 2018). Several studies have illustrated the functions of FMRFamide-like peptides (*flp*) in both *C. elegans* (Atkinson et al., 2016; Lee et al., 2017; Nelson et al., 2014) and plant-parasitic nematodes, including cyst (You et al., 2021; Atkinson et al., 2013) and root-knot nematodes (Hada et al., 2020; Johnston et al., 2010; Kumari et al., 2017). These peptides show diversity in their expression and function in behavioral regulation. Knocking down some of these *flps* via RNAi reduced the infection ability of the nematodes. This evidence demonstrates the potential of targeting nematode nervous systems to achieve control aims.

Neurotransmitters are small molecules that are important regulators of animal behaviors (Chase and Koelle, 2007). In *C. elegans,* neurotransmitters have been extensively studied. However, less is known about the localization and function of neurotransmitters in plant-parasitic nematodes. γ-Aminobutyric acid (GABA) is the most prominent inhibitory neurotransmitter in *C. elegans.* GABA-immunoreactive neurons are found primarily in motor neurons in *C. elegans* (Jorgensen, 2005; Gendrel et al., 2016).

Our previous studies showed that nematodes display greater variation in the number of motor neurons than previously expected (Han et al., 2015), and we found a reduction of GABA-immunoreactive neurons during the development of the cyst nematode *Heterodera glycines*, which is associated with the loss of mobility of this nematode (Han et al., 2018). However, little information is available for GABA and GABA-immunoreactive neurons in stylet-bearing nematodes.

Here, we examine the GABA-immunoreactive neurons in several additional stylet-bearing nematodes, including the economically important root-knot and root-lesion nematodes. Using pharmaceutical assays, we also investigate the role of GABA signaling in the locomotion behaviors of these nematodes.

## Materials and Methods

### Nematode cultures

*Meloidogyne incognita* was originally isolated from soybean plants (gift of Dr. Jason Bond) and cultured on susceptible tomato plants in the greenhouse. *Pratylenchus penetrans* was extracted from monoxenic corn root cultures (Lauritis et al., 1983). Seeds for monoxenic cultures were surface sterilized and germinated on water agar. Post-germination, roots were extracted and transferred to Gamborg's agar along with *P. penetrans* (Gamborg et al., 1968; Rebois and Huettel, 1986). *H. glycines* was maintained on susceptible soybean plants (cv. Macon) grown in sandy loam soil in a laboratory growth chamber. The fungal feeder *Aphelenchus avenae* was extracted from cultures maintained on ¼ strength potato dextrose agar with the fungus *Botrytis cinerea*, as previously described by De Soyza (1973).

*P. penetrans* and *A. avenae* were extracted from Petri dish cultures using water submersion, while *M. incognita* was extracted from blended plant root tissues using the sugar centrifugation method (Jenkins, 1964). All extracted nematodes were washed three times with distilled water before fixation.

### Immunohistochemistry

Nematodes were extracted and washed three times with water. The fixation procedure followed a previously described method (Gendrel et al., 2016) with modifications (Han et al., 2018). Nematodes were incubated in a solution of 4% paraformaldehyde and 2.5% glutaraldehyde at 4 °C for 15 min and washed 3 times with PBST (8 mM Na_2_HPO_4_, 150 mM NaCl, 2 mM KH_2_PO_4_, 3 mM KCl, 0.05% Triton X-100, pH 7.4). Nematodes were segmented with a razor blade to increase their permeability (Hussey, 1989). Segments were then incubated in 100 mM Tris/1mM CaCl_2_ and 2 mg/ml proteinase K for 20 min at room temperature. After washing three times with PBST, segments were incubated in pre-chilled methanol on ice for one minute followed by one minute in pre-chilled acetone. The segments were then washed three times with PBST again. The segments were blocked in PBST/1% BSA for at least 1 hour followed by incubation in a 1:100 anti-GABA at 4 °C overnight.

The day after washing with PBST, the segments were incubated in a 1:100 secondary antibody PBST/0.1% BSA at 4 °C overnight. Segments were washed again in PBST before observation. Nematodes were mounted onto microscope slides with 2% agar pads, and images were taken using a Zeiss M2 Axio Imager with mechanized stage and Zeiss Zen software (Zeiss Microscopy Solutions, Jena, Germany) using both differential interference contrast and fluorescent filters. Z-projections were created and analyzed using ImageJ. Over 100 individuals with positive staining were examined.

### GABA agonist behavioral assays

Nematodes were extracted, washed in sterile water, and concentrated to a 1 mL volume of 0.01% Triton-X 100 in water. All chemicals tested were dissolved in water. For piperazine (Sigma-Aldrich, Cat. No. P45907), 1, 10, 20, 50 and 100 mM were tested along with 0.01% Triton-X as control. For GABA (Sigma-Aldrich, Cat. No. A2129), 1, 10, 50, and 100 mM were tested along with 0.01% Triton-X as control. The concentration of piperazine was originally chosen based on a previous study (Miltsch et al., 2013); however, the stylet-bearing nematodes appeared to be less sensitive to GABA than *C. elegans*, and thus a series of higher concentrations of the compound were used in this assay compared to what would be used with *C. elegans* (McIntire et al., 1993).

Nematodes were incubated in 10 mL of each concentration for 10 minutes before rating their movement. The percentage of nematodes with kinked necks and paralysis was calculated. At least 30 nematodes were examined for each compound with a given concentration. Each experiment was performed twice. The number of nematodes that exhibited the hook shape or were completely paralyzed was recorded. These data were grouped to display the overall percentage of nematodes affected at varying concentrations of the compounds. Data were analyzed using the software Prism 6.0 (GraphPad, San Diego, California, USA) to obtain the EC_50_ values.

### GABA antagonist picrotoxin behavioral assays

Picrotoxin is insoluble in water, and was thus dissolved in dimethyl sulfoxide (DMSO). In order to minimize the potential toxic effects of DMSO on nematodes, a 100mM solution of picrotoxin in DMSO was diluted down to 5% DMSO and 5mM picrotoxin (Sigma-Aldrich, Cat. No. R284556). A control with 5% DMSO in water was used. Five nematodes were incubated for 5 minutes in the treatment or control before being hand-picked into the center of a 1-cm diameter circle on a water agar plate. After 20 minutes on the plate, nematodes were observed to be either inside or outside the drawn circle. Three replications of this procedure were performed per experiment, with two experiments per species. The concentration of picrotoxin was kept consistent throughout experiments. At the end of the assay, the number of nematodes that were able to move outside the circle was recorded and a comparison was made using Student's unpaired t-test for significance between the treated and control groups.

## Results

### All examined nematode species have GABA-immunoreactive neurons

**Figure 1:**
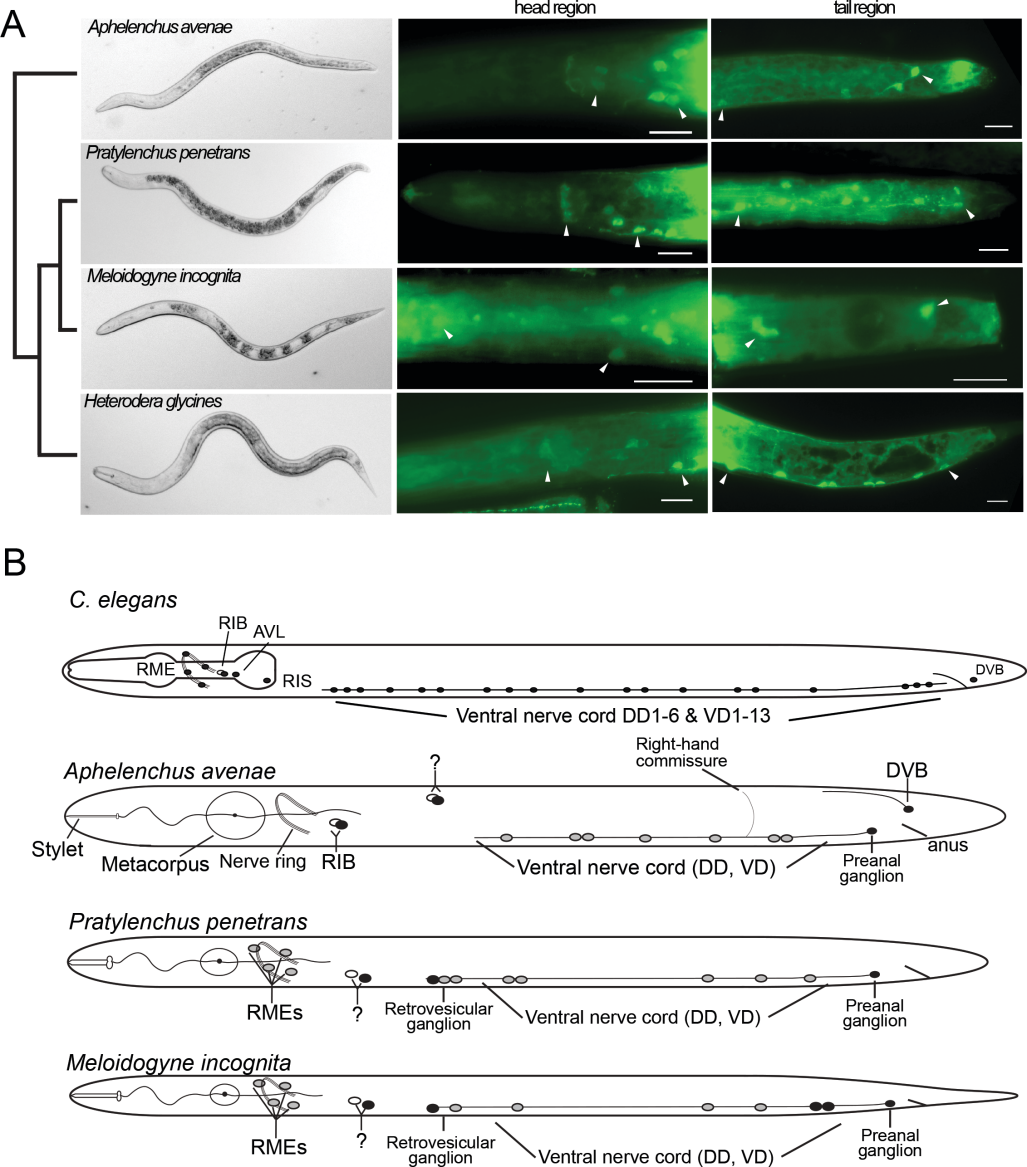
The GABA nervous system in stylet-bearing nematodes. 1A. The staining patterns of the GABA-immunoreactive cells in *Aphelenchus avenae*, *Pratylenchus penetrans*, *Meloidogyne incognita* and *Heterodera glycines*. From the left, the first column shows the light-microscopy images of the examined species. The second and third columns show the antibody staining of the neurotransmitter GABA in the head and tail regions. Arrowheads indicate GABA-immunoreactive neurons. Scale bar = 10 μm. B. The illustration of GABA-immunoreactive neurons that were detected using anti-GABA staining at a given stage in examined nematode species. All nematodes are displayed with anterior to the left and the dorsal side up. Neuron symbols with black filling are strongly stained cells, while gray filling indicates more weakly stained neurons. The staining patterns of the adult *Aphenlenchus avenae* female, adult *Pratylenchus penetrans* male, and *Meloidogyne incognita* second stage juveniles (*Heterodera glycines* second stage juveniles is from our previous study Han et al., 2018). Neuronal homologs are labeled based on the knowledge of *C. elegans* (Gendrel et al., 2016). GABA-immune positive cells found in *C. elegans* with either weak staining or considered involved in GABA clearance are not shown in the illustration. Illustration does not reflect true size.

The immunohistochemistry procedures confirmed the presence of GABA-immunoreactive neurons within the head, ventral nerve cord, and tail in all examined species (Fig. 1A). We observed variability in the staining pattern between each species. This variability includes instances where staining was observed in a position without a known homologous GABAergic neuron in *C. elegans.* We also observed substantial variability among individuals of the same species.

In *A. avenae* heads, we observed consistent staining in a bilateral pair of neurons positioned 15–20 μm posterior of the nerve ring on the ventral side of the lateral ganglion. The only similarly located pair of GABA-immunoreactive neurons in *C. elegans* are the RIB neurons (Figs. 1B; Gendrel et al., 2016). Additionally, the fungal feeder *A. avenae* contains a bilateral subdorsal pair of GABA-positive neurons near the anterior dorsal cord. GABAergic neurons in *C. elegans* have not been reported from that position. In *C. elegans,* the non-neuronal head mesodermal cell (HMC) is located in a similar position and was shown to stain with GABA antibody (Gendrel et al., 2016), likely playing a role in clearance of GABA. Interestingly, HMC is born during embryogenesis as a pair, but one of the pairs undergoes programmed cell death prior to hatching.

In both *M. incognita* and *P. penetrans,* a subventrally positioned pair of neurons was consistently located approximately 25 μm posterior of the nerve ring (Figs. 1A&B). The right neuron of this pair was always located slightly (~ 1 μm) anterior of its left pair. It isn’t clear whether this asymmetrical pair are homologs of the RIB neurons or present a novel cell type. The *C. elegans* RME neurons are four GABAergic motor neurons that surround the nerve ring at dorsal, ventral, and lateral positions. While RME homologs were found in *H. glycines* (Han et al., 2018), we observed only inconsistent staining in likely RME homologs from the species tested here.

GABAergic staining in the tail of *C. elegans* hermaphrodites is limited to the single DVB neuron located in the dorsal-rectal ganglion and three motor neurons in the preanal ganglion (Hall and Russell, 1991). A GABA-immunoreactive neuron was routinely found in *A. avenae* within the dorsal-rectal ganglion, indicating homology with DVB (Figs. 1A&B). However, unlike in *C. elegans*, this putative DVB homolog sends a process into the dorsal cord rather than to the pre-anal ganglion and anteriorly along the ventral nerve cord. We did not observe GABA-immunoreactive cells in the dorsal-rectal ganglia of the other nematodes examined. Similarly, each nematode studied was observed to have only one GABA-immunoreactive neuron in its preanal ganglion.

We found multiple GABA-immunoreactive neurons in the ventral nerve cord (VNC) of every species, indicating its role in regulating locomotion (Figs. 1A&B). The retrovesicular ganglion, which includes several motor neurons that can be considered part of the VNC, contained one or more immunoreactive neurons in *M. incognita* and *P. penetrans*.

We observed two types of spacing between GABA-immunoreactive VNC neurons. First, in all species, we would occasionally find cell bodies that were immediately adjacent to each other with no obvious neuron in between them. In *C. elegans*, the VD and DD classes of neurons are GABAergic and, in several instances, immediately adjacent to one another. More frequently, individual neurons in our species were found spaced at regular intervals. For example, we found a very regular repeating pattern of one VNC neuron every 15 μm in *A. avenae.*

Commissures from the ventral to dorsal cord were only observed in *A. avenae.* These commissures were exclusively found on the right side. This is similar to *C. elegans,* but differs from our findings in *H. glycines*, where the majority of VNC commissures were found on the left. Due to a lack of a whole-mount antibody staining method in stylet-bearing nematodes, we were not able to compare numbers of GABA-immunoreactive neurons across species. Nevertheless, our data indicate variation in the GABA nervous system that could contribute to the regulation of the behaviors of these nematodes.

### Disrupting GABA signaling can affect locomotion behaviors

**Figure 2:**
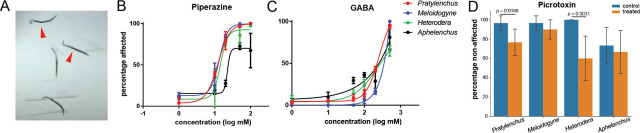
Behavioral assays in response to GABA and GABA antagonists (A). Characteristic “hook shape” *Heterodera glycines* second-stage juveniles (marked by red arrowheads) induced via disrupting GABA signaling. The half-maximal effective concentration (EC_50_) of paralysis caused by piperazine (B) and GABA (C). Piperazine or GABA was dissolved in water with 0.01% Triton-X. Water with 0.01% Triton-X was used as control. Active nematodes were incubated in 10 ml of the chemical at each concentration for 10 min on a petri dish before observation. At a given concentration of each chemical, more than 30 individuals from a species were tested and the experiment was performed twice. Data were pooled from both experiments. Nematodes showing a hook shape or complete paralysis were counted as being affected (D). The locomotion assay of picrotoxin. Five nematodes of a given species were treated with 100 mM picrotoxin dissolved in 5% DMSO for 5 min and then were hand-picked on to the center of a 1-cm diameter circle on a water agar plate. After 20 min, nematodes outside the circle were counted, and the ratio of this number to the total number of test nematodes was calculated

In *C. elegans*, GABA has both inhibitory and excitatory functions (Jorgensen, 2005). Thus, we further investigated how GABA signaling affects locomotion behaviors in these nematode species using GABA agonists and an antagonist. GABA, and the GABA agonist piperazine, caused the nematodes in our assays to develop a characteristic “hook shape” that progressed to a complete flaccid paralysis (Fig. 2A). Through the assays with different concentrations, we determined the concentration that caused half of the population to become hook-shaped and paralyzed. For piperazine, *P. penetrans*, *M. incognita*, and *H. glycines* had similar EC_50_ values, which were 12.3, 13.4, and 14.4 mM, respectively, while *A. avenae* had an EC_50_ value of 22.5 mM (Fig. 2B). In comparison, the EC_50_ values to GABA were 240 and 264 mM for *P. penetrans* and *H. glycines*, respectively (Fig. 2C), and the EC_50_ values for both M. incognita and *A. avenae* were over 10,000 mM, suggesting these two species were insensitive to exogenously-applied GABA (Fig. 2C).

The GABA antagonist picrotoxin was shown to cause a loss of locomotion in *M. incognita* (Wright et al. 1984). We found that in *P. penetrans* and *H. glycines*, the effect of picrotoxin significantly hindered nematode movement, with *p*-values of 0.0169 and 0.0031, respectively. However, picrotoxin did not affect *A. avenae* or *M. incognita*, yielding *p*-values of 0.6191 and 0.2596, respectively (Fig. 2D).

## Discussion

Tylenchomorpha in clade 12 contains plant-parasitic nematodes of economic importance (van Megen et al., 2009). Relatively few studies have explored the GABA-immunoreactive system in stylet-bearing species (Stewart et al., 1994; Wright et al., 1984). Despite their relatively close phylogenetic relationship, the different species we examined showed substantial variation in their GABA-immunoreactive systems among themselves and in comparison to *C. elegans* and *Ascaris* (McIntire et al., 1993; Guastella et al., 1991). In addition, we found GABA-immunoreactive neurons with no obvious homolog in *C. elegans* (e.g., neurons found on the dorsal side in *A. avenae*). However, our data cannot determine whether this is due to the absence of these neurons or a change to the neurotransmitter identity.

Together, these data support our previous finding that variation exists in the neuroanatomy in nematode species (Han et al., 2015). We were able to observe possible consistent patterns, as well as definite differences, between the GABA-immunoreactive neurons. However, our methods did not allow us to identify and specifically characterize individual neuron types for each species. In addition, antibody staining of GABA also labels cells that take up and/or recycle GABA, and so may not be solely an indicator of GABAergic neurons (Gendrel et al., 2016).

The examined species have different parasitic behaviors and responses to their environment (Perry, 1996; Wyss and Grundler, 1992; Zunke, 1990). The variation in their GABA-immunoreactive systems may, in part, contribute to this feature. For example, root-knot nematodes lose their mobility during development, while the closely related root-lesion nematodes do not. We previously found that a reduction in the number of GABA-immunoreactive neurons is associated with the loss of mobility in the soybean cyst nematode (Han et al., 2018). Further research into understanding this idea would be useful for pinpointing the regulation of movement in nematodes.

We also found differences in the behavioral response to compounds affecting GABA signaling among species. Specifically, *A. avenae* was found to be affected very differently compared to other species. In the test with piperazine, *A. avenae* did not reach the EC_50_ until a high concentration. These results may be due to the permeability of piperazine to *A. avenae*, or variation in the structure or number of GABA receptors in *A. avenae* in comparison to the other species being tested. Similarly, *A. avenae* was not obviously affected by picrotoxin. This lack of difference suggests that the GABA antagonist does not bind and block the GABA receptors as severely in *A. avenae* as it does in other species.

One downside of this study is that in plant-parasitic nematodes, in order to perform antibody staining, the nematodes have to be segmented (Stewart et al., 1994). It was therefore impossible to develop a complete map of GABA-immunoreactive cells or a confident enumeration of the number of VNC GABA-immunoreactive neurons. A second potentially confounding variable is lack of specificity for some compounds. For example, picrotoxin is cross reactive with other ligand gated channels (Liu et al., 1994).

GABA is the prominent inhibitory neurotransmitter in *C. elegans* and regulates behaviors, including moving and foraging (McIntire et al., 1993). GABA has been found in both plant- and animal-parasitic nematodes (Johnson and Stretton, 1987; Guastella et al., 1991; Stewart et al., 1994). Our results suggest that GABA is present in Clade 12 stylet-bearing nematode species and likely has a functional role.

However, different from previous results (Wright et al., 1984), we did not detect any effect from picrotoxin in *M. incognita*. This might be due to two factors. First, the picrotoxin was dissolved differently in Wright et al. (1984). Second, those authors measured the undulation of the treated nematodes, while we measured the distance the nematodes moved. It will be interesting to look further into the use of GABA in *A. avenae* specifically and determine why or how the receptors may be regulated differently than in other species. These results suggest that GABA is involved in the regulation of locomotion behavior. Disruption in GABA signaling could cause defects in locomotion behaviors, which would reduce infection and subsequent reproduction. Therefore, studying the GABA system in plant-parasitic nematodes could potentially provide new, more specific targets for their control.

Neurotransmitters and their corresponding signaling pathways are most likely conserved in animals. We previously demonstrated that serotonin also regulates feeding and reproductive behaviors in the root-lesion nematode *P. penetrans* (Han et al., 2017). However, the receptors of neurotransmitters might display a high level of diversity, which can be potentially used to find novel and more specific targets for control. One recent study has shown that the GABA receptors from the root-knot nematode to be potential pharmaceutical targets for control (Nomura et al., 2021). Thus, a better understanding of the nervous system of stylet-bearing nematodes may improve our control strategies.
